# Not only the seed matters: Farmers’ perceptions of sources for banana planting materials in Uganda

**DOI:** 10.1177/0030727020930731

**Published:** 2020-06-22

**Authors:** Fleur BM Kilwinger, Pricilla Marimo, Anne M Rietveld, Conny JM Almekinders, Ynte K van Dam

**Affiliations:** 1 Knowledge Technology and Innovation Group, Wageningen University and Research, Wageningen, the Netherlands; 2 Bioversity International, Kampala, Uganda; 3 Consumer and Marketing Studies, Wageningen University and Research, Wageningen, the Netherlands; 4 CGIAR Research Project on Roots, Tubers and Bananas, Montpellier, France

**Keywords:** Adoption, means-end chain analysis, repertory grid analysis, seed sources, seed sourcing strategies, seed system

## Abstract

The adoption of improved seed and other planting material in developing countries shows mixed results despite their potential to increase agricultural productivity. To arrive at a better understanding of the observed adoption rates, a lot of research is focused on finding the cultivars and variety traits that are attractive to farmers. Given smallholder farmers’ seed sourcing practices are often influenced by social ties and cultural norms, it is also relevant to understand where and why farmers seek to acquire planting material. In this study, means-end chain analysis was applied to understand farmers’ perceptions of formal and informal sources of banana planting material. Means-end chain analysis allows respondents to select and verbalize their own constructs to evaluate a product or service. These personally relevant constructs are subsequently linked to their personal goals via laddering interviews. We interviewed 31 Ugandan banana farmers from Western and Central region. Farmers associated formal sources mainly with improved cultivars, tissue culture plantlets and low levels of diversity. Informal seed sources were mostly associated with traditional cultivars, suckers and high levels of diversity. The goals farmers pursued while acquiring planting material, such as financial gains, food security, and to sustain and develop the household, were fairly similar among different groups of farmers. The means through which farmers aimed and preferred to pursue these goals differed and could be related to aspects such as gender, production scale and production goals. These differences among farmers preferences for particular sources indicate that not only cultivar traits should be tailored to farmers’ preferences and needs, but also the characteristics of the sources from which farmers access planting material.

## Introduction

Improved agricultural technologies promoted by governments and other actors are not necessarily adopted by farmers, particularly in developing countries (Almekinders et al., 2019b; Walker and Alwang, 2015). This might be explained by a lack of information and understanding on farmers’ preferences and priorities and the way the improved technologies fit their realities (Almekinders et al., 2019b). In line with this, it is argued that agricultural innovations should not be viewed as stand-alone technological improvements but rather as elements of an agricultural innovation system which includes social elements as well (Klerkx et al., 2012). An improved agricultural technology might be considered beneficial because of its potential to increase yield, but the real-life outcome of adopting the technology by farmers might be variable due to non-technological elements, such as culture, personal preferences, and institutional arrangements.

Many technology development efforts in agriculture deal with the improvement of planting material, particularly in the form of breeding improved cultivars and improving propagation methods. However, much less research goes into understanding how technologies, i.e. seeds (true seeds and other propagation materials^1^) of improved cultivars, can be accessed and how this differs between farmers. Evaluating the sources and delivery channels of planting material of vegetative propagated crops such as potato, cassava or banana is especially important because the material is usually bulky, highly perishable, difficult to store, has low production rates compared to “true seed crops,” and is prone to easy build-up of pathogens that affect seed health (Bentley et al., 2018).

In developing countries, informal (local, traditional, farmer) seed systems are the dominant sources of planting material for vegetatively propagated crops (Almekinders et al., 2019a; Andrade-Piedra et al., 2016). Seed exchange among farmers is usually strongly influenced by social ties and cultural norms, rarely involves monetary transactions, and provides farmers with planting material of cultivars adapted to their agro-ecological and socioeconomic conditions (e.g., Adam et al., 2018; Kilwinger et al., 2019a; McGuire, 2008, Tadesse et al., 2017; Van Niekerk and Wynberg, 2017). Formal seed systems, in contrast, are characterized by the production and distribution of tested seed and registered improved cultivars, following strict quality control measures (Almekinders et al., 1994).

On-farm seed multiplication and exchange can result in the build-up and transfer of diseases (e.g., Andrade-Piedra et al., 2016; Jacobsen et al., 2019; Thomas-Sharma et al., 2016). For example, the spread of Banana Xanthomonas Wilt (BXW) in Uganda has partially been attributed to exchange of infected planting material among farmers (Blomme et al., 2014; Karamura et al., 2008; Kubiriba and Tushemereirwe, 2014). In some regions of Uganda where highly susceptible cultivars dominated, the rapid spread of BXW wiped out entire banana groves (Rietveld et al., 2014; Tinzaara et al., 2013). To prevent these kinds of disasters, numerous seed system interventions aim at providing farmers with clean and disease resistant planting material. This is usually done by establishing and strengthening the formal seed system.

One of the larger recent interventions in the banana seed system in Uganda was the Tissue Culture (TC) program by, among others, the National Agricultural Research Organization (NARO), Bioversity International and the International Institute of Tropical Agriculture (IITA) (Kikulwe, 2016). This project aimed to make TC banana plantlets available to farmers via improved market pathways, private partnerships and improved institutional policies. Considerable effort went into the establishment of demonstration trials and nurseries to familiarize farmers with the use of TC banana plantlets; normally farmers plant banana suckers (e.g. Kilwinger et al., 2019b). Research findings demonstrated the superior performance and profitability of TC plantlets over regular banana suckers (e.g., Kabunga et al., 2012a; Kikulwe, 2016). The plausible reaction from farmers following such initiatives would be adoption, but despite efforts and the presumed benefits, use of TC plantlets among Ugandan farmers remained relatively low. Sales of TC plantlets at nurseries dropped seriously after the project ended and some nursery owners even mentioned a decline in sales of up to 70% (Kilwinger et al., 2017). The explanation for such a situation tends to be found in the performance of the materials being supplied (e.g. Kabunga et al., 2012a), economic factors hindering adoption (e.g. Murongo et al., 2019; Muyanga, 2009) and other technology-acceptance factors (Mulugo et al., 2019). The type and characteristics of the source or provider of the materials is usually not considered.

Formal sources, as compared to informal sources, may not only offer different cultivars and types of planting material but also the procedure of acquiring the material is likely to be different. Unlike informal sources, formal sources often involve transport costs, (higher) cash requirements, and no social relation is developed between the buyer and seller. Little is known about how such differences in the seed sourcing procedure influence farmers’ decision and choice for a particular seed source. It is therefore important to isolate beneficial from inconvenient differences as well as to assess the effect of these differences. In addition, it is relevant to understand how these benefits and inconveniences play out for different types of farmers: characteristics of the household or farmer like sex, level of education and farming experience, as well as household farm size, income, and the relative importance of banana production as compared to other livelihood activities can play a role in determining seed needs, preferences and purchasing power. If, for example, formal sources have large volumes of planting material available, this might be a beneficial characteristic for large-scale farmers but irrelevant for small-scale and subsistence farmers who often require smaller quantities.

In this paper, we apply the means-end chain analysis to understand how farmers perceive banana planting material from different sources including private sector companies, public organizations, nongovernmental organizations, and local sources such as neighbors and the own farm. The means-end chain analysis was developed in the 1980s to understand how consumers evaluate, and why consumers value the products or services they purchase (Grunert and Grunert, 1995; Gutman, 1982). The method acknowledges individual differences in experiencing reality by allowing respondents to select and verbalize their own constructs by which their reality is linked to their personal goals (Reynolds and Gutman, 1988; Walker and Olson, 1991). This makes means-end chain analysis a valuable tool for cross-cultural and cross-subcultural studies (e.g., Barrena et al., 2015; Valette-Florence, 1998). Recently, the means-end chain method has been used to understand farmers’ perceptions of agricultural technologies and practices (e.g. Hansson and Lagerkvist, 2015; Ngigi et al., 2018; Okello et al., 2018; Tey et al., 2015; Urrea-Hernandez et al., 2016). In this study we further explore the usefulness of this method for the identification of delivery conditions of banana planting material that are attractive to farmers.

## Methods

### Study areas

The study was conducted in two districts in Uganda: Mukono in the central region and Mbarara in the western region of the country. The districts were chosen based on differences in cultivation history, intensity of banana production and level of activity of formal seed system actors. In Central Uganda, banana is a traditional crop which has been cultivated for hundreds of years (Rietveld and Farnworth, 2018). Due to diseases, low soil fertility and labor constraints, production in Central Uganda declined over the last three decennia and shifted to western parts of the country were banana cultivation is relatively new (Bagamba et al., 2010, Gold et al., 1999). As a result, banana production in Western Uganda is more intensive and commercial whereas in Central Uganda, production goals are more focused towards home consumption and traditional uses (Kilwinger et al., 2019a). According to the 2009/10 agricultural census report (UBOS, 2010), the western region had the largest production of cooking banana (68%) followed by the central region (23%). The promotion of improved planting material by nongovernmental organizations (NGOs) and government institutes was more intense in selected areas of the Mukono district in Central Uganda as compared to Mbarara district in Western Uganda. The promotion of TC banana also started in the central region of Uganda in 2008 (Kikulwe, 2016).

### Study design

Farmers from the study sites in Central and Western Uganda were selected via quota sampling. The research team moved around in the chosen villages to encounter sufficient farmers willing to participate while keeping in mind the need to select a diverse group of respondents in terms of sex, age and farm size. In total, 32 farmers—16 from each district—participated in the means-end chain analysis. In Mbarara district, one interview could not be completed, hence it was dropped from analysis. Demographic information on age, sex, total farm size and area under banana production was collected from each respondent. In addition, farmers were asked about general aspects of their banana production, the seed sources and cultivars they used, and whether they had been beneficiaries of banana seed system interventions. Farm households that estimated that they cultivated banana on an area larger than 1.6 ha were classified as large-scale farmers. Prior to data collection, five enumerators, three men and two women, had received a 2-day training on the interview technique.

After collecting the demographic and banana production characteristics of the household, means-end chain interviews were conducted. The interviews consisted of two parts: attribute elicitation and laddering. The elicitation technique we used was triadic sorting based on Kelly’s repertory grid. In this technique, the respondent is presented with consecutive triplets of three fairly similar products or services which have to be sorted according to similarities and differences perceived by the respondent (Kelly, 1955). In our study, farmers were presented with triplets of cards which had sources for banana planting material written on them in the local language. In total, farmers were presented with nine cards each with a different seed source, including five formal and four informal sources. The sources were a laboratory, a nursery, the National Agricultural Advisory Services (NAADS), the National Agricultural Research Organization (NARO), a nongovernmental organization (NGO), a large-scale farmer, a remote farmer, a neighbor and own farm (Table 1).

**Table 1.** Brief description of the nine seed sources for banana planting material used in the study.

	Source	Description
Formal	Laboratory	A laboratory producing tissue culture (TC) banana plantlets. Tissue culture plantlets are produced in laboratories and can be distributed on behalf of other organizations and to nurseries, but can also directly be accessed by farmers (Kilwinger et al., 2017). Sourcing from a laboratory meant farmers directly acquired the planting material from the laboratory without any intermediate organization or nursery.
	Nursery	A nursery for banana planting material. Several nurseries have been established as part of seed system interventions (Kikulwe, 2016). Nurseries usually provide TC plantlets but since most nurseries have a large mother garden, suckers can also be obtained.
	National Agricultural Advisory Services (NAADS)	The National Agricultural Advisory Services (NAADS) is a public agency responsible for agricultural advisory/extension services. One of NAADS’ programs was the distribution of banana planting material, either in the form TC, corms or suckers
	National Agricultural Research Organisation (NARO)	NARO mainly develops, but sometimes distributes new banana cultivars either in the form of TC, corms or suckers (Kilwinger, 2017).
	Nongovernmental organization (NGO)	Some NGOs such as Caritas distribute banana planting material among their members, either in the form of TC, corms or suckers (Kilwinger et al., 2017).
Informal	Large-scale farmer	A large-scale banana farmer within the community.
	Remote farmer	A banana farmer from outside the community. Farmers mainly exchange banana suckers within the community but exchange with farmers from other communities also occurs (Kilwinger et al., 2019b).
	Neighbor	A neighboring farmer. Farmers often refer to fellow farmers within the community as neighbors even if they are also relatives or friends and not direct neighbors (Kilwinger et al., 2019b).
	Own farm	The own farm. In both districts around 70% of the suckers is sourced from the own farm (Kilwinger et al., 2019b).

When all seed sources were discussed with the farmer, (s)he was presented with nine predefined triplets of cards (full data presentation underlying the reported results in this article are available in Kilwinger et al., 2020). In case a farmer was not familiar with a particular source, all the sets including that particular source were removed. Each time the farmers were presented with a triplet of cards they were asked to group two sources which, according to them, appeared to be more similar as opposed to t other. While doing so the farmers were given the following scenario:

“Imagine you have to source banana planting material for the coming planting season. I now present you with three seed sources where you could source this planting material. Which two seed sources have, according to you, more similarities as opposed to the other?”

After grouping the seed sources, respondents were asked to describe *why these two were similar compared to the other one*, resulting in a list of constructs and contrasts also called “bipolar word-pairs.” From each set of triplets, the sources which were grouped together were noted with the related constructs. When all the triplets were presented and the word-pairs listed, farmers were asked to indicate for each bipolar word-pair, which of the two features they preferred when sourcing banana planting material. Further responses were elicited using a soft-laddering approach. In this free response format, respondents construct ladders with personally meaningful constructs (Phillips and Reynolds, 2009). Soft laddering is the recommended technique in studies with a relatively small sample size (<50) and of an exploratory nature (Costa et al., 2004). The starting points of the laddering was the preferred feature, i.e. the preferred construct of each bipolar word-pairs listed during the elicitation phase. From each preferred construct a series of “*Why is it important to you that…*” questions were asked. Through asking, a ladder of constructs was created starting from attributes to perceived consequences and personal values. It was emphasized to the respondents that there were no right or wrong answers and that the aim of the interview was to understand their individual preferences.

### Analysis

The elicited word-pairs and ladders were coded individually by two researches and thereafter compared and merged. In cases of inconsistencies, the researchers discussed and agreed which code was most suitable using original interview transcripts. Coded responses were categorized into attributes, consequences and values. Thereafter, an implication matrix was constructed to count the number of respondents making direct and indirect links between constructs. The implication matrix was constructed manually using spreadsheet software. From the implication matrix, an overall hierarchical value map (HVM) was constructed showing the links between constructs by transforming individual ladders into chains. A cutoff level of four was chosen for the HVM which means that only links which were mentioned by four or more (≥13%) respondents were shown. The cutoff level was based on the principle of showing as much links as possible while still remaining with a clearly interpretable HVM (Grunert and Grunert, 1995). Indirect, nonredundant, links were also presented in the HVM if they we mentioned by six or more (≥19%) respondents. Separate HVMs were created by grouping farmers according to district, production scale (large–small) and sex (male–female). Group sizes for each of these categories differed hence a different cutoff level for each HVM was chosen, aiming to represent chains established by minimally around 20% of the farmers.

## Results

### Characteristics of the interviewed farmers

In total, 17 men and 14 women were interviewed (Table 2). The total farm size of the interviewed farmers ranged from 0.2 ha to 65 ha with an average of 8.2 ha. In both areas, men reported larger farms and more farm area cultivated with banana than women. Total farm size and area under banana cultivation was larger in the western region (12.2 and 2 ha) than in central (3.9 and 0.6 ha) which resulted in more western farmers being classified as large-scale farmers. In general, about half (48%) of the farmers indicated that they grow improved or introduced cultivars such as FHIA hybrids, Yangambi KM5 and M9. The use of improved cultivars was higher in the western region compared to central (68% and 38% respectively). In both areas, more men reported growing improved cultivars compared to women as well as more large-scale farmers compared to small-scale farmers. More farmers in the central region, men and large-scale farmers, had been beneficiaries of previous banana seed system interventions

**Table 2.** Demographic characteristics of the respondents and banana production characteristics of the household per region, sex and farm size.

	**All (*N* = 31)**	**Central (*n* = 16)**	**Western (*n* = 15)**	**Men (*n* = 17)**	**Women (*n* = 14)**	**Large scale (*n* = 11)**	**Small scale (*n* = 20)**
Age (yrs.) (SD)	42.6 (13.8)	41.1 (13.2)	42.6 (13.8)	43.8 (15.3)	39.3 (9.8)	40.4 (12.7)	42.6 (13.6)
Total farm size (ha) (SD)	8.2 (15.1)	3.9 (7.2)	12.2 (19.3)	11.2 (17.6)	4.5 (10.9)	19.7 (20.0)	1.1 (1.0)
Banana farm size (ha) (SD)	1.3 (1.9)	0.6 (0.6)	2.0 (2.3)	1.7 (2.3)	0.8 (0.9)	3.1 (2.5)	0.5 (0.4)
Uses improved cultivars (%)	48.4%	37.5%	60.0%	64.7%	28.6%	72.7%	35.0%
Beneficiary of intervention (%)	19.4%	25.0%	13.3%	23.5%	14.3%	27.3%	15.0%

SD: standard deviations.

### Farmers’ perceptions of banana seed sources

Farmers were not familiar with all the presented seed sources. In both regions, respondents were least familiar with laboratories (9 out of 31) followed by NGOs and nurseries (11 and 14 out of 31 respectively). The formal source known to most farmers was NAADS (27 out of 31). Farmers in the Western region were less familiar with formal sources. Almost all farmers were familiar with informal sources: only two female farmers from the Western region mentioned that they did not know any remote farmer they could source planting material from.

Farmers mentioned a total of 24 different bipolar word-pairs during the elicitation phase (Table 3). The number of elicited word-pairs per respondent ranged between 2 and 11 with an average of 7. The most frequently mentioned constructs and contrasts were cultivar related. “Traditional cultivars” and “improved cultivars” were mentioned most often by farmers. “Traditional cultivars” were mainly associated with informal sources and “improved cultivars” with formal sources. The cultivar related word-pair thereafter named most frequently were availability of “other cultivars” and “similar cultivars.” With “other cultivars,” farmers meant the source provided cultivars which they did not have on their own farms whereas “similar cultivars” meant the source had cultivars they were already growing on their plantation. “Other cultivars” were associated with both formal and informal sources. The formal source most associated with “other cultivars” was NAADS and a remote farmer and a large-scale farmer were the most related informal sources. “Similar cultivars” were mainly associated with informal sources and most often with the own farm. Another cultivar related word-pair was a source with a “high cultivar diversity” available and a “low cultivar diversity.” A source with “high cultivar diversity” was mostly related to informal sources. Farmers were also considering whether they could be “sure of the cultivar type,” which they related to both formal and informal sources.

**Table 3.** The constructs and contrast elicited during triatic sort and the number of times farmers related them to a formal or informal seed source (*n* = 31).

	Formal sources^‡^					Informal sources					Formal sources					Informal sources			
Constructs^†^	LB	NS	NA	NR	NG	LF	RF	NE	OF	Contrasts	LB	NS	NA	NR	NG	LF	RF	NE	OF
Traditional cultivars	–	1	2	1	–	10	8	26	14	Improved cultivars	1	3	15	13	5	5	–	–	–
Similar cultivars	–	–	1	–	–	9	3	9	17	Other cultivars	–	3	8	4	1	9	12	3	–
Close	–	1	–	–	–	8	3	13	13	Far	2	3	7	7	2	6	9	–	–
Unknowledgeable	–	–	–	1	1	2	9	10	7	Knowledgeable	–	1	2	6	2	11	4	2	–
Suckers	–	–	2	–	–	2	5	8	7	TC	3	8	9	7	3	–	–	–	–
Diseases	–	1	3	2	–	–	–	8	8	Disease free	2	6	2	9	1	8	–	–	–
Informal	–	–	–	–	–	4	4	8	3	Formal	5	7	7	9	1	–	–	–	–
Free of charge	–	–	8	–	–	–	4	–	7	Pay cash	4	6	4	3	2	7	–	–	–
Not use STI	–	–	–	–	–	–	9	10	7	Uses STI	1	1	2	6	1	–	–	–	–
Small quantities	–	–	2	–	1	2	4	5	7	Large quantities	3	4	–	–	–	5	2	–	1
Cheap	–	–	–	–	–	–	4	4	–	Expensive	4	6	4	3	2	7	–	–	–
Unsure of cultivar	–	–	2	2	–	–	2	3	–	Sure of cultivar	–	4	1	5	1	6	2	–	6
High cultivar div.	–	–	1	1	–	5	3	3	4	Low cultivar div.	–	1	2	1	–	1	–	2	2
Assessable	–	–	–	–	–	1	1	3	5	Not assessable	–	–	1	1	–	5	2	2	–
Exchange	–	–	–	–	–	3	3	8	3	No exchange	–	1	–	–	–	3	–	–	–
On demand	1	2	–	1	–	1	2	5	3	At their convenience	–	–	3	–	–	–	1	–	–
Low input req.	–	–	–	1	–	3	–	5	1	High input req.	1	2	2	2	–	1	–	–	–
Low quality	–	–	1	2	1	1	–	2	1	High quality	1	1	–	1	–	2	1	–	1
Adapted argo-eco	–	1	–	2	–	1	1	3	3	Not adapted agro-eco	–	–	–	–	–	2	1	–	–
Low resource av.	–	–	–	–	–	–	3	–	2	High resource av.	–	–	–	–	–	6	–	–	–
No disease resistance	1	1	2	1	2	–	–	–	1	Disease resistance	–	–	–	–	–	–	1	1	1
No terms/conditions	–	–	–	–	–	1	2	2	–	Terms/conditions	–	–	4	1	2	–	–	–	–
Familiar	–	–	–	–	–	–	–	1	2	Unfamiliar	1	2	2	1	–	–	–	–	–
Trusted	–	1	–	–	–	–	–	–	–	Not trusted	–	1	–	1	–	–	–	–	–

^†^ Which attribute in the word-pair is the construct and which the contrast differs per respondent. For ease of interpretation of each word-pair one is presented in this table as the construct and one as the contrast.

^‡^LB = laboratory, NS = nursery, NA = NAADS, NR = NARO, NG = NGO, LF = large-scale farmer, RF = remote farmer, NE = neighbor, OF = own farm.

Apart from the cultivars available at the source, an important feature for farmers was whether “suckers” or “tissue culture plantlets” were available. “Tissue culture plantlets” were only related to formal sources and “suckers” to informal sources and NAADS. Other word-pairs related to the planting material available at the source were if the material was “free of diseases” or “diseased,” whether there was a “high quantity” available or a “low quantity,” if managing the material required a “high resource input” or a “low resource input,” if the material was “adapted to agro-ecological” conditions or not, and if the material was “disease resistant” or not.

Next to word-pairs related to the planting material, farmers made constructs and contrasts based on the acquisition procedure. The most frequently mentioned word-pair was a source “located close” and one “located far away.” Informal sources were mostly perceived as “close by” and formal sources, a remote farmer, and a large-scale farmer as sources “located far away.” Another frequently mentioned word-pair was whether the source was “knowledgeable” or “unknowledgeable.” A “knowledgeable source” was described as a source where farmers could obtain additional advice on proper management of the planting material and their banana plantation in general. Large-scale farmers and formal sources were mostly perceived as a “knowledgeable” source whereas neighbors were perceived as “unknowledgeable.” Farmers also mentioned a “cash payment” requirement or if the planting material could be obtained “free of charge” via “exchange,” if the material was “expensive” or “cheap,” if the source was “innovative,” if the source was “familiar” to them and if certain “terms and conditions” needed to be met while acquiring the material. With “terms and conditions” farmers meant the material could not be obtained “on demand” when they need it. Instead, the acquisition procedure involved “terms and conditions” such as subscription requirements, farm inspections, a limited quantity and no free choice in cultivar type. Formal sources were mostly related to “cash requirements,” “expensive,” “innovative,” “unfamiliar” and involving “terms and conditions.” Attributes that can be related to seed system intervention such as meeting terms and conditions, the type of planting material available and a cash requirement were more frequently mentioned by farmers from the study site in Central Uganda compared to Western Uganda.

### Relating attributes, consequences and values while selecting a seed source

**Figure 1.**
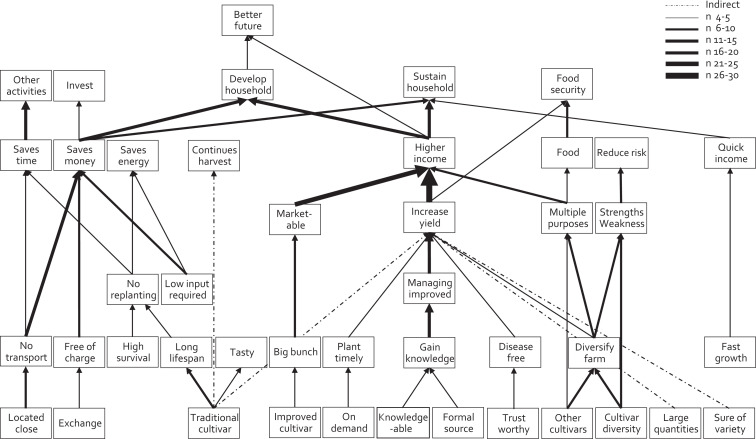
Hierarchical value map based on the number of respondents making a link between constructs. The thickness of the arrow correlates with the number of respondents making a link. Nonredundant, indirect, links between constructs are presented with a dashed line. *n* = 31; cutoff level *n* = 4.

The number of ladders constructed per farmer ranged between 3 and 36 with an average of 16. In the HVM, 42 constructs appear, which is 47% of the total named constructs. Between the constructs, 51 direct links are shown representing 12% of the total number of direct links made between constructs (Figure 1). The construct mentioned by most farmers was “higher income.” Farmers said it would be used to “sustain” and “develop” the household and have a “better future.” The majority of farmers said a high income resulted from “increased yield,” “marketable products,” and products which could be used for “multiple purposes.” An increased yield was mostly related to “improved management” after farmers had “gained knowledge.” An increased yield was also related, by fewer farmers, to “disease free” planting material and “timely planting.” Most farmers attributed marketability to a “big bunch.” With “multiple purposes” the farmers meant the produce could be used for income, food and other purposes leading to “food security.” Products with multiple purposes resulted from having a farm with “diverse cultivars.” Most farmers linked this to a source with a “high cultivar diversity” or “other cultivars.” Other benefits of a farm with diverse cultivars were “risk avoidance,” because each cultivar has its own “strengths and weaknesses.”

Farmers mentioned other financial gains besides increasing the income. They also took into consideration how the money would come into the household and made a distinction between “higher income” meaning more income is generated, “saving money” by not having to spend money, “quick money” meaning a relatively large sum is obtained in a short time, and a “continuous flow of money.” Saving money was mostly linked to similar values as a high income but resulted from different consequences such as “free” planting material, “no transport” requirement and cultivars with “low input requirements.” These were in turn linked to attributes which were mainly related to informal sources such as nonmonetary “exchange” of planting material and a source in a “nearby location.” Besides saving money, it was important for farmers to “save time” and “save energy” which were also mainly linked to attributes related to informal seed sources. Farmers valued saving time because this allowed them to do “other activities” besides farming.

#### Differences in hierarchical value maps of Central and Western Uganda

**Figure 2.**
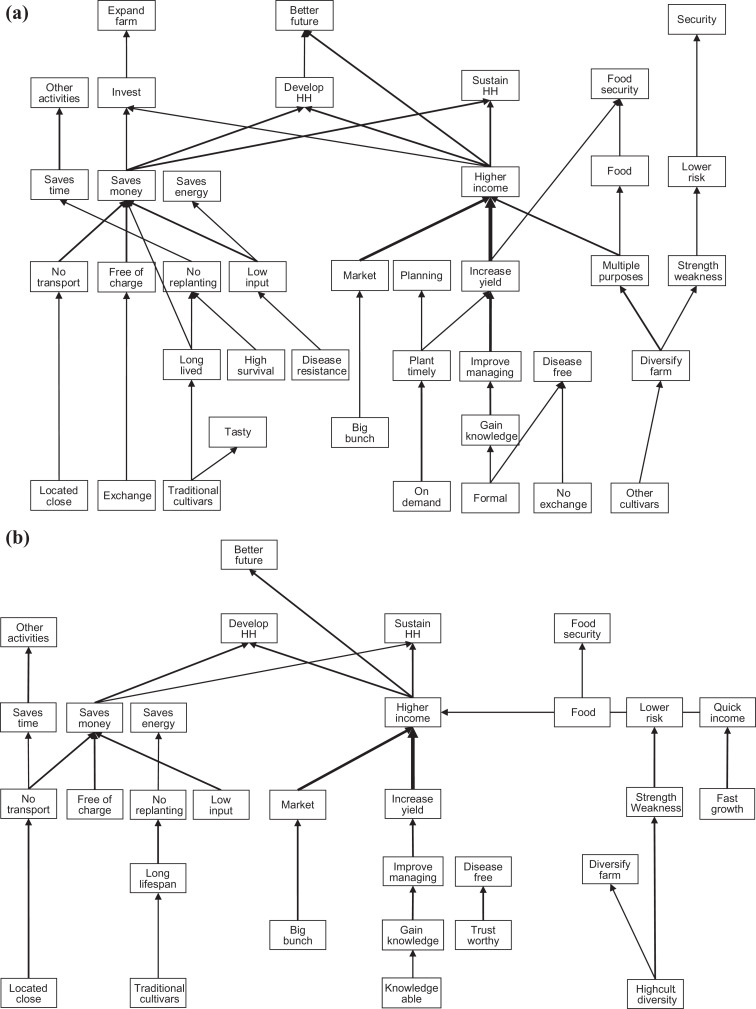
Hierarchical value map based on the number of respondents making a link between constructs of the study site in the (a) central region (*n* = 16; cutoff level *n* = 3) and (b) western region (*n* = 15; cutoff level *n* = 3).

Many chains in the HVMs of Central and Western Uganda were overlapping but the HVM of Central presented more links (Figure 2). The most dominant pathway in the overall HVM—”gaining knowledge” to “high income”—was represented in the HVMs of both areas. Gaining knowledge was related to “formal” sources by farmers from Central and to a “knowledgeable farmer” by Western farmers. In the HVM of Central, the bipolar constructs “exchange” and “no exchange” of planting material appeared whereas in the HVM of Western none of the two appeared. Farmers from Central Uganda preferred sources that exchange planting material because it is “free”; and sources that do not exchange planting material because the material is more likely to be “disease-free.” They also associated disease-free planting material to formal sources. Western farmers related disease-free planting material to “trustworthy sources.” “Disease resistance” only appeared on the HVM of Central but was not sufficiently linked to a single attribute reaching above the cutoff level. Another chain that was represented only in the HVM of Central Uganda was planting material that can be obtained “on demand” which enables farmers to “plant timely” leading to higher yields. In the HVMs of both Central and Western Uganda, a “diversified farm” appeared. In Central, farmers related a diverse farm to “multiple purposes” and “avoiding risks.” In Western a diversified farm was linked to risk avoidance only. The relation between “traditional cultivars” and a “tasty” product only appeared in the HVM in Central, whereas “fast growing” planting material and “quick income” only appeared in the HVM of Western.

#### Differences in hierarchical value maps large-scale and small-scale farmers

Similar to the HVM of Central and Western Uganda, there were a lot of overlaps between the HVMs of the large and small-scale farmers (Annex 1). The main difference was that the HVM of the large-scale farmers contained almost twice as many links as that of small-scale farmers. The most dominant chain in the overall HVM, from “gaining knowledge” to “higher income,” was represented in both the HVMs. Large-scale farmers linked gaining knowledge to “formal” sources and sources who put a lot of effort in “innovation” whereas small-scale farmers related it to a “knowledgeable” farmer. Gaining knowledge was linked to a source that is innovative only in the HVM of large-scale famers. Two other chains that appeared only on the HVM of large-scale farmers, which were also not present in the overall HVM, were improved cultivars linked to “disease resistance” and “tissue culture (TC)” planting material linked to “disease-free” planting material. Large-scale farmers associated disease-free planting material with “formal” and “trusted” sources. The chain from “on demand” to “plant timely” was only represented in the HVM of the large-scale farmers. Chains that only appeared on the HVM of small-scale farmers and were absent on the HVM of large-scale farmers were “traditional cultivars” for their “long lifespan,” and free “exchange” of planting material because there was no monetary cost. Attributes appearing in the HVM of large-scale farmers such as “innovative,” “TC plantlets,” “improved cultivars,” and “formal” were linked mainly to formal sources (Table 3). Large-scale farmers mentioned more values compared to small-scale farmers. All the values that appeared in the HVM of small-scale farmers also appeared in the one for large-scale farmers. In addition, large-scale farmers constructed chains from making “investments” to “expanding the farm” and from “higher income” to “self-direction” and “status.”

**Annex 1.**
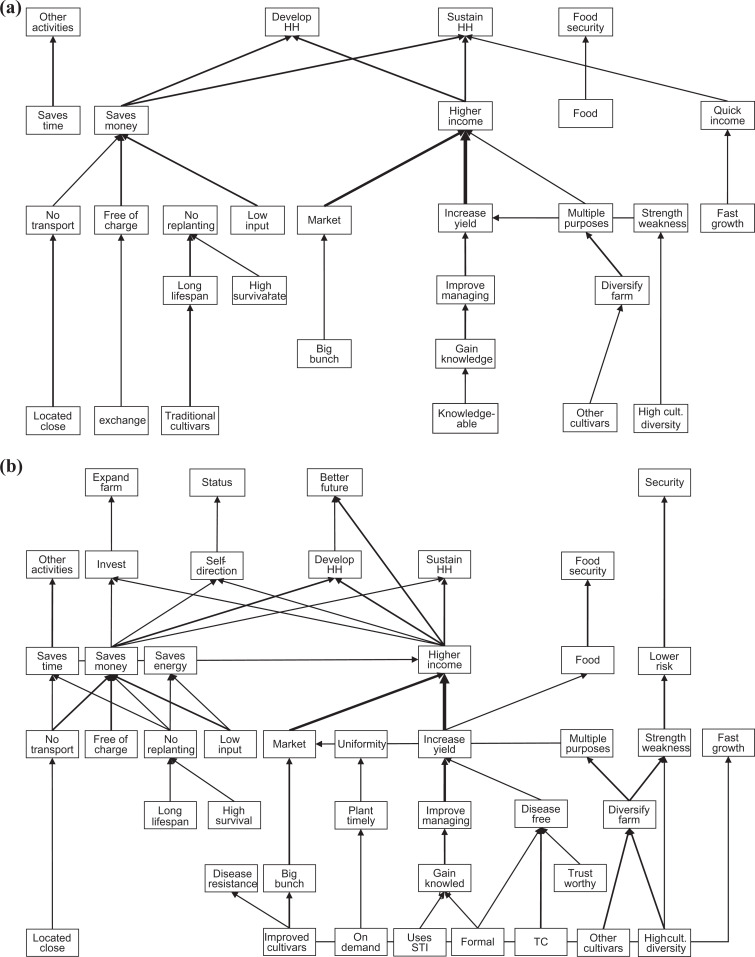
Hierarchical value map based on the number of respondents making a link between constructs of a) small-scale farmers (cutoff level *n* = 4; *n* = 20) and b) large-scale farmers (cutoff level *n* = 2; *n* = 11).

#### Differences in hierarchical value maps between men and women

In the HVM of both men and women, the most dominant chain from “gain knowledge” to “high income” was present but there were differences in the related attributes: men linked gaining knowledge to “formal” sources and women to a “knowledgeable” farmer (Annex 2). The largest difference between the HVMs was that men created chains from both “improved” and “traditional” cultivars whereas in the HVM of women only “traditional cultivars” appeared. Men preferred improved cultivars because of their “big bunches,” “fast growth” and for providing “quick income.” Both men and women associated traditional cultivars with a “long lifespan” which is valued because it requires “less replanting.” Men also valued traditional cultivars because they are “adapted to agro-ecological” conditions in their fields and therefore yield big bunches. The link between “time saving” and “other activities” appeared in both HVMs but was mentioned more often by women compared to men. In addition, the chain “on demand” and “plant timely” was only present in the HVM of the women. Both men and women pursued similar values such as food security and, sustaining and developing the household.

**Annex 2.**
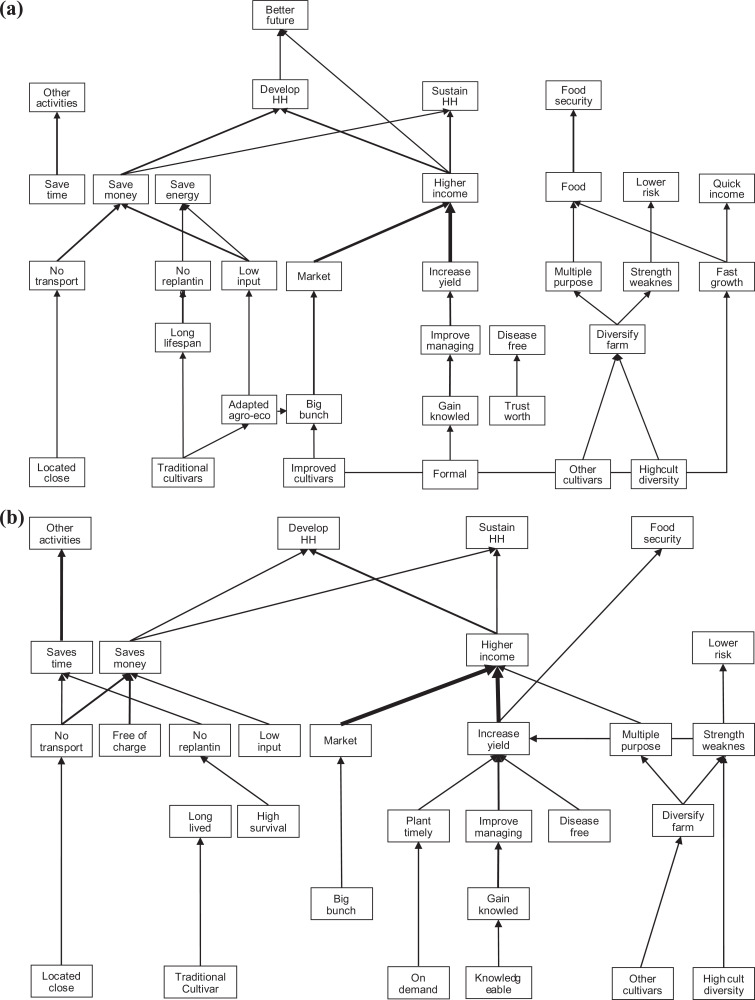
Hierarchical value map based on the number of respondents making a link between constructs of a) men (*n* = 17; cutoff level *n* = 3) and b) women (*n* = 14; cutoff level *n* = 3).

## Discussion

### Source characteristics

The results show that when selecting a source for banana planting material, farmers take more attributes into consideration than only the type of planting material available. Farmers also considered diversity of available cultivars, the chances of finding new (“other”) cultivars, quantities of planting materials available and the timing of the availability. Although source characteristics related to the available planting material were most frequently mentioned, farmers also considered knowledge availability, transportation requirements, trustworthiness and transaction conditions when choosing seed sources. The majority of the identified attributes have been described and discussed in literature (e.g. Sperling, 2002; Kabunga et al., 2012a; Murongo et al., 2019; Muyanga, 2009). Yet, some of the attributes, especially the ones in the social domain and related to diversity, are seldom described.

How farmers related attributes to sources differed among, but also within, formal and informal sources. For example, NAADS—like the informal sources—was perceived as a free source, whereas other formal seed sources were not. Large-scale farmers had many overlapping attributes with formal sources such as “knowledgeable,” “sure of cultivar” and “disease free,” but were also perceived by some farmers as “inaccessible” and “expensive.” This supports the claim that within informal seed networks, seed does not just move fluidly between farmers without barriers and at minimal cost (Coomes et al., 2015). Seed sources, either formal or informal, not only differ in the seed they have available, but also their acquisition procedures, and thus attractiveness. These factors beyond the performance of the material can facilitate or hinder purchase and adoption. Seed in this way is not a fixed entity; it is reconstructed and reconfigured as it is handled by different actors (Glover et al., 2019). It also supports notions that seed systems are similar to innovation systems and as such harbor complex interactions between social and technical components (Glover et al., 2019; McGuire 2008).

### Pursued benefits

When sourcing planting material, farmers pursued more benefits and goals than merely an increase in yield and income. Farmers looked for planting material that could be used for multiple purposes, required less time and labor to manage and that would reduce risks. These other benefits and goals were mainly related to traditional cultivars and a high cultivar diversity, which in turn were mostly associated with informal seed sources. Formal sources in collaboration with informal sector could therefore ensure that they have necessary diversity demanded by farmers given the values that farmers associate with a diverse portfolio of cultivars.

Farmers did not only point out that financial gain is important, they also indicated the importance of the amount, timing and frequency of these gains. Attributes related to informal sources such as exchanging planting material and no transport requirement were mainly valued because they lead to a reduction in expenditure—i.e. they saved money, whereas attributes related to formal sources, such as big bunches and clean planting material, were mainly valued because they generated income. Planting material from different sources can thus result in different types of financial gains. For example, availability of large quantities of planting material of a single cultivar can lead to a large and uniform harvest over a short time span, resulting in a large sum of money at once (quick income). Having a high cultivar diversity on the other hand can lead to staggered harvest times and thus, a continuous harvest and smaller but continuous amounts of cash income. In the field of development economics, this is referred to as an “income smoothing mechanism” (Morduch, 1995). Income smoothing mechanisms used by rural households in developing countries include e.g. labor diversification within the household, crop diversification and migration (Barrett et al., 2001; Pellegrini and Tasciotti, 2014). The results of this study suggest that banana cultivar diversification is another mechanism used by farmers for income smoothening, risk avoidance and food security. Continuous harvest and income were mentioned by one-third of the farmers but were not sufficiently linked to other constructs to appear in the hierarchical value map. There was no specific group of farmers that mentioned these constructs which explains why they also did not appear on the grouped HVMs. Preferences in income distribution might differ among farmer or household typologies and change over time. In some periods, farmers might need more income, for example during the time when school fees have to be paid. School fees, classified under sustaining the household in this research, was frequently mentioned by farmers. During the time when school fees have to be paid, farmers might also prefer to source planting material from their own farm and save money over buying planting material.

### Different pathways to shared values

The HVMs derived from different groups of farmers showed many similarities, especially at the values level. This suggests that farmers pursue similar goals but identify different pathways to reach these goals. For example, the pathway from gaining knowledge to a higher income was most dominant and represented in all HVMs. Where farmers seek this knowledge differed per group. Farmers from Central Uganda, large-scale farmers and men perceived formal sources as an important place to obtain knowledge whereas farmers from Western Uganda, small-scale farmers and women more often perceived a knowledgeable fellow farmer as a source to obtain knowledge. Not all formal sources were perceived as knowledgeable. Providing knowledge next to planting material itself seems to be important to make a source attractive to farmers. Access to knowledge was found to be an important factor for adoption of TC plantlets (Kabunga et al., 2012b). Large-scale farmers, frequently referred to as knowledgeable in this study, may provide an important role for farmers in the community that cannot directly access information from formal actors.

Observed differences between the study sites in Central and Western can be related to seed system interventions, cultivation history and production objectives. Attributes related to formal seed sources, which farmers usually get familiar during interventions, were mentioned more often by farmers from Central Uganda. Farmers from Central Uganda valued high cultivar diversity because of the multiple purposes of banana, which seems less important to farmers from Western. Multiple purposes, meant banana products could be sold and used in various ways, indicating emphasis on both marketing and home use. The appreciation of large-scale farmers for attributes related to formal sources of planting material points to their commercial interests, but at the same time the HVMs show that they also appreciate benefits from attributes related to informal sources. Large-scale farmers have not dropped the traditional use of banana as a multipurpose livelihood product, meaning they are in a way “dualistic”: they maintain the profile of a traditional smallholder farmers and are adding considerations that are typical for a commercial larger farmer with interests in economic gains. The overlap between large-scale farmers and men can explained by the fact that large-scale farmers were more often male and suggests men are more market-oriented than women, which is also found by Rietveld et al (2020). The market-orientation is related to valuing improved cultivars for their big, marketable bunches and for their fast growth leading to quick income. Small-scale farmers and women on the other hand perceived more benefits from traditional cultivars. Women valued time availability for other activities more than men, possibly because women have multiple chores in the household and could have their own crop priorities (Kasente et al., 2002).

## Conclusion

The means-end chain analysis has provided insights in how different types of farmers perceive various sources of banana planting material and why they value them. The use of triatic sort and soft laddering approach allowed us to capture farmers” considerations while avoiding preselection and predefinition of any attributes. This resulted in answers which might not easily emerge in survey-based data collection. The importance of obtaining knowledge while sourcing planting material was striking, in combination with the finding that larger and male farmers considered the formal sources to obtain knowledge, whereas smaller farmers and women saw more opportunity to obtain knowledge from informal sources. Another finding was that not only the amount of income generated is important to farmers, but also the timing and frequency of incomes.

The availability of diverse cultivar types is a very important attribute of an attractive source of banana planting material to all types of farmers but in addition farmers considered many aspects of seed sources which are unrelated to the type of cultivar or planting material. These included the location of the source, the transaction type, the availability of knowledge, trustworthiness, the time planting material is available, and required labor and time investments to manage the planting material. Thus farmers do not merely look for clean and high yielding planting material that can increase income but take more characteristics related to of the source and the planting material in consideration. The goals farmers pursued while sourcing banana planting material were mainly overlapping. The attributes and consequences farmers presumed would lead them to these goals differed among farmers. For example, some farmers” strategy to sustain the household was use of free planting material of traditional varieties that would save them money whereas other famers invested in improved varieties that generate more income.

In this paper we described the results of a case study on farmers” perceptions of banana seed sources. Due to the relatively small sample size, sampling strategy, and the limited information on this topic yet available, we cannot make any claims about the external validity and generalizability of the outcomes. What we can conclude is that among the interviewed farmers not only seed, but also seed sources, matter, and that farmers have diverging perceptions on the attractiveness of these source when seeking new planting materials. This is an important consideration for seed system interventions. In the case of introduction of tissue culture banana plantlets, it means that failure of adoption is not necessarily found in the performance of the technology itself. Tissue culture plantlets were mainly available at formal seed sources or distributed as part of government programs. Formal sources are not equally attractive/accessible for all farmers and involve a rather different acquisition procedure. Careful consideration of the sources at which improved planting material is made available could improve seed system interventions.

In general, we identified that perceived benefits and disadvantages of seed sources differ among farmers. Understanding these differences in preferences among farmers is relevant for seed system interventions in order to strategize on seed delivery pathways. Aggregation of this type of information could result in the definition of “delivery profiles”: these would not only comprise cultivar traits and client profiles that breeders seek to suit different farmer typologies (Ashby and Polar, 2019)—but would also include contextual agro-ecological and socioeconomic variables which facilitate accessibility of the planting material. Such “delivery profiles” would be of strategic importance to projects that aim to reach differentiated groups of farmers with new cultivars, clean planting material and disease management.

## Note

1. Vegetative propagated crops like banana are not multiplied via “seeds” in the botanical sense of the word. Because of the absence of a one-word term for the non-seed organs of plants that are used to reproduce crops they are referred to as “planting material,” “vegetative seed,” or simply “seed” (Andrade-Piedra et al., 2016). Farmers can use several types of banana planting material such as suckers, tissue culture plantlets and corms (Jacobsen et al., 2019). We use the terms “seed” and “planting material” interchangeably to refer to its propagates and “suckers” and “TC plantlets” when appropriate.
